# Feelings are Messy: The Feelings We Study in Affective Science Should Be Too

**DOI:** 10.1007/s42761-024-00263-z

**Published:** 2024-09-05

**Authors:** Anthony G. Vaccaro

**Affiliations:** Department of Psychology, University of Southern California, Los Angeles, CA USA

**Keywords:** Emotion measurement, Emotion theory, Valence, Research methods, Mixed emotions

## Abstract

Affective science has taken up the challenge of building a bridge between basic affective science and practical applications. The articles in the Future of Affective Science issue lay out methodological and conceptual frameworks that allow us to expand affective science into real-world settings and to handle naturalistic methods. Along with these advances, accomplishing this goal will require additionally refocusing the types of experiences we study, and the measures of experience we are interested in. This paper explores the necessity for basic affective science to embrace the messy and complex nature of human emotion in order to bridge the gap between theoretical concepts and real-world applicability. Specifically, this involves studying experiences that do not fit as neatly into dominant conceptual frameworks, such as valenced scales and the most common discrete emotion categories, and that may be more difficult to measure or experimentally control. This makes the gap between affective science and real-world feelings larger. To move the field towards incorporating emotional complexity in an empirical manner, I propose measurement standards that err on the side of less fixed-choice options and using stimuli chosen for their potential to elicit highly complex responses over time within the same individual. Designing studies that can measure these experiences will push emotion theories to explain data they were not originally designed for, likely leading to refinement and collaboration. These approaches will help capture the full spectrum of human emotional experience, leading to a more nuanced and applicable understanding of affective science.

Why are you interested in affective science? Even without knowing who is reading this paper currently, I can guarantee it is not because of how organized, systematic, and controlled the experience of emotion is.

Affective science now aims to be applicable to real-world settings and to more accurately reflect the spectrum of experiences across individuals. Throughout the special issues on the Future of Affective Science, researchers grapple with the gap between basic affective science and real-world applicability, presenting new methods and conceptual frameworks for bridging this divide (Shiota et al., 2023). Specifically, many highlight the importance of real-world and naturalistic emotion measurement (Hoemann et al., 2023; Lin et al., 2023; Rocklin et al., 2023; Tran et al., 2023) and the need to acknowledge influences which have been traditionally absent from basic affective science, such as interpersonal effects on emotion (Petrova & Gross, 2023), cultural variation in experience and norms (Brady et al., 2023; Vishkin & Tamir, 2023; Yu et al., 2023), and the temporal dynamics of affect (Lange 2023; Mukherjee et al., 2023). An important step in bridging the current divide between basic affective science and real-world practicality is to make paradigms in basic affective science more naturalistic, reflecting these types of influences. However, if we want to study more natural feelings, we also have to dig deeper into why the current gap between basic affective science and real-world translation is as large as it is. *The measurements we use, and studies we design, only allow us to study varieties of affect that fit neatly into expectations from discrete and constructionist theories.* These basic and univalent states represent only a fraction of our experience, and moving affective science forward requires studying “messier” feelings.

## What are “Messy” Feelings?

In referring to feelings, I use the definition of the subjective experience of affect (Adolphs, 2017). But what makes a feeling messy? I use the term “messy” to emphasize feelings, and qualities of feelings, that would not be captured by standard valence-arousal or discrete emotion measures.

**Fig. 1:**
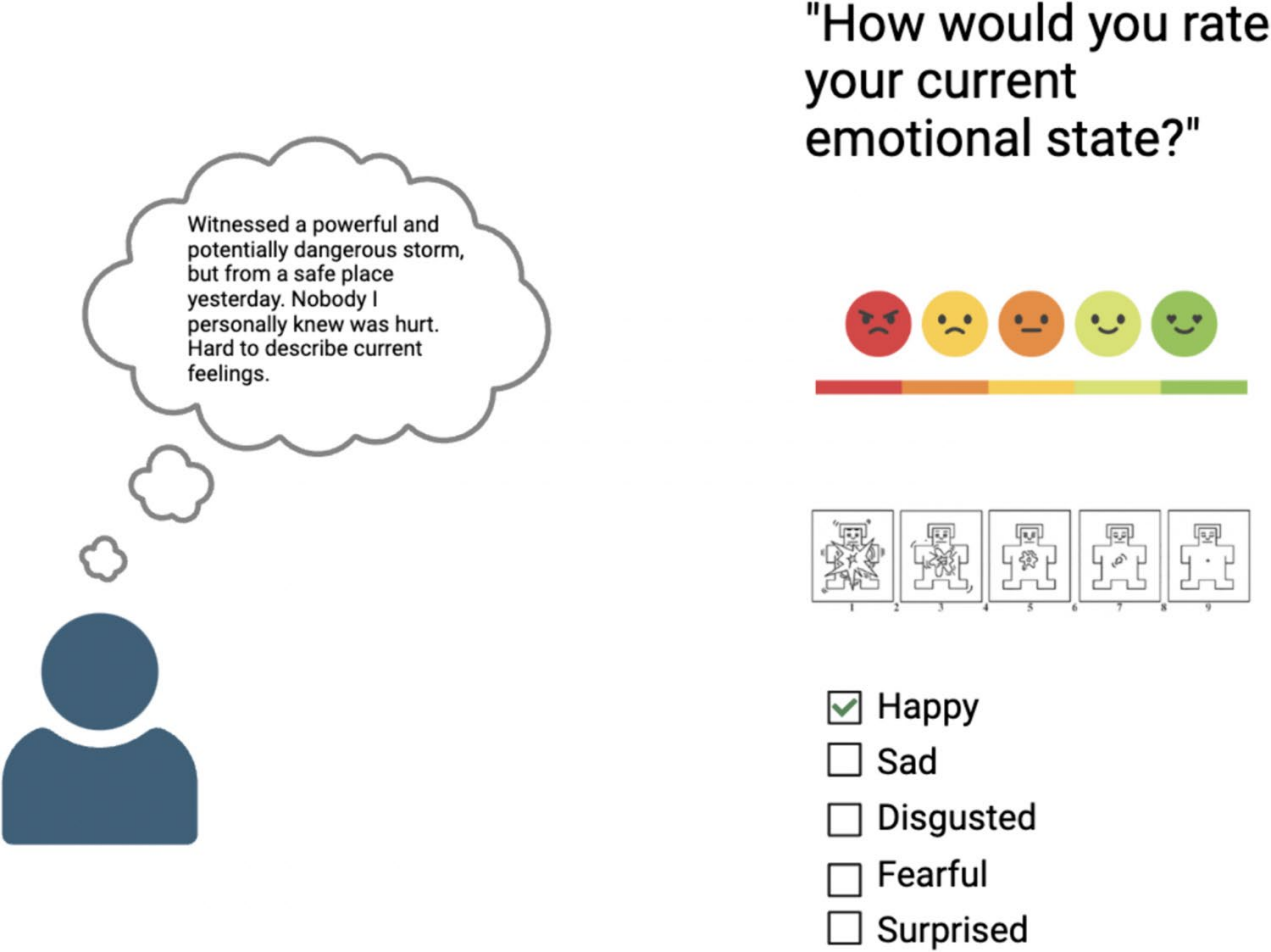
Common forced-choice measures of affect may erase essential information. Real-world affect can entail multiple valences, atypical states such as awe and relief, blends between emotion categories, and can be colored by participants feeling certain or uncertain of how they currently feel. These are critical sources of variance in experience that have to be removed to report experience in the most common methods of measurement. Figure created with BioRender.com

When we measure feelings, there are states and dimensions of affect our measures capture well, as that is what they are optimized to do (Mauss & Robinson, 2010). A video of a snake lunging to bite you is intended to induce a state that discrete would be labelled as “fear” on a checklist of common discrete emotions, or that would be rated as negatively valenced with high arousal. We choose this stimulus because of its reliability across subjects in inducing a specific, unambiguous, univalent affective experience. However, when we apply these same measures to real-world experience, we fail to account for states that may not be commonly captured by either categorical or valence-arousal scales and for potential blends or switches in emotions. Additionally, state-level attitudes towards affect, such as uncertainty of what one is feeling, color the experience. When our common fixed-choice measures are applied to real-world settings where non-univalent or blended feelings occur, we end up with data representing a subject’s best attempt to reduce their current experience into overly narrow siloes. While all affective measurement is, on some level, reducing complex experience into a simplified quantitative measure, the measures we most commonly miss are essential sources of variance in experience (Fig. 1).

## These Measures Filter the Topics We Choose to Study

As Walle and Dukes (2023) discuss, valence is a feature of affect we rarely critique or act cautiously about, and yet their well-presented evidence shows that it is itself poorly operationalized and not a concept affective scientists agree about. The dimension of valence is treated in such a manner that researchers can feel limited in their ability to research experiences that do not neatly fit into a valenced framework. Gasper’s (2023) article discusses how even an experience as common as neutrality can be overlooked when affect is only considered in terms of positivity vs. negativity. Mixed feelings such as bittersweetness, longing, and awe (Larsen & McGraw, 2014) and states with unclear or variable valence such as surprise (Mellers et al., 2013; Neta & Kim, 2023) remain understudied. There are also clinically relevant emotional reports such as “feeling nothing,” or the sense of “not knowing how you feel,” which are well studied in the context of depersonalization and alexithymia respectively—but these trait level measures imply relevant dimensions of feeling on the state level, such as uncertainty or non-differentiation (Aaron et al., 2018; Brewer, et al., 2016; Kashdan et al., 2015; Roberts, 2019; Simeon, et al., 2003).

The status of these states as emotions, feelings, or attitudes has been questioned due to their lack of obvious fit, which also results in their under investigation. Part of overcoming this requires exploring more potential measurement dimensions for an affective experience such as ambiguity, automaticity, or state-level measures of emotional awareness (Bailen et al., 2019; Brainerd, 2018; Neta et al., 2018; Smith et al., 2019; Wielgopolan & Imbir, 2024). However, beyond developing and targeting novel measurement dimensions, there are simple changes to the methodological standards of our field that can naturally expand our studies to capture greater emotional complexity.

## Change 1: Promoting More Flexible Measurement to Capture Messier States

A researcher studying events that trigger awe or bittersweetness will quickly understand that bipolar valence and arousal scales, or a forced-choice check-list of discrete states, are not enough to generate adequate data (Berrios et al., 2015; Shiota et al., 2007; Yaden et al., 2019). However, the adjustments needed to study these states should not be limited to those actively choosing to study messier experiences. As we build larger collaborative datasets, our measurement should err towards more flexibility and less forced-choices on the part of participants. At minimum, this involves asking for both positive *and* negative valence ratings separately and allowing subjects to report as many categories of discrete emotion as they want. While a subject experiencing a univalent or distinct state can still report that, forced-choices on valence or discrete categories permanently reduce data for subjects experiencing something more complex. For researchers with strong interests in discrete states or a theoretical argument against blended states, we can still ask subjects for the most prominent state, and those who are more interested in univalent judgements can still calculate these values. Additionally, incorporating a short free-response box when interested in categories of emotion can provide richer data. Even if rarely used by the subject in certain studies, this allows participants to describe their emotional experiences in their own words, capturing nuances that fixed-choice measures might miss. Additionally, we gain further confidence in understanding what our stimuli are inducing in individual subjects.

## Change 2: Emphasizing Intrasubject Variability in Lab Studies

Lab emotion studies have been moving towards using more naturalistic stimuli, which aim to better capture relevant aspects of emotion such as temporal change and broader context (Lange, 2023; Mukherjee et al., 2023; Saarimäki, 2021). This has been a crucial start to making lab studies more relevant to real-world contexts, as the concrete and moderately evocative stimuli once used by affective science have clear limits in ecological validity (Shiota et al., 2023). My proposal is that we can go further in their use to increase their value. Firstly, we should be encouraged to not assume the emotion category or valence being induced without collecting subject-specific reported data—a scenario that commonly occurs in fMRI paradigms specifically. In my own work using film stimuli to induce transitions between positive, negative, and mixed feelings, I found that for most of the film, only 40–70% of subjects were in consensus at any given moment about which of the states they were feeling. Crucially, using subjects’ individually reported feeling timecourses led to vastly different results for our fMRI analyses than attempting to define segments of the movie by emotional consensus (Vaccaro et al., 2024).

**Fig. 2:**
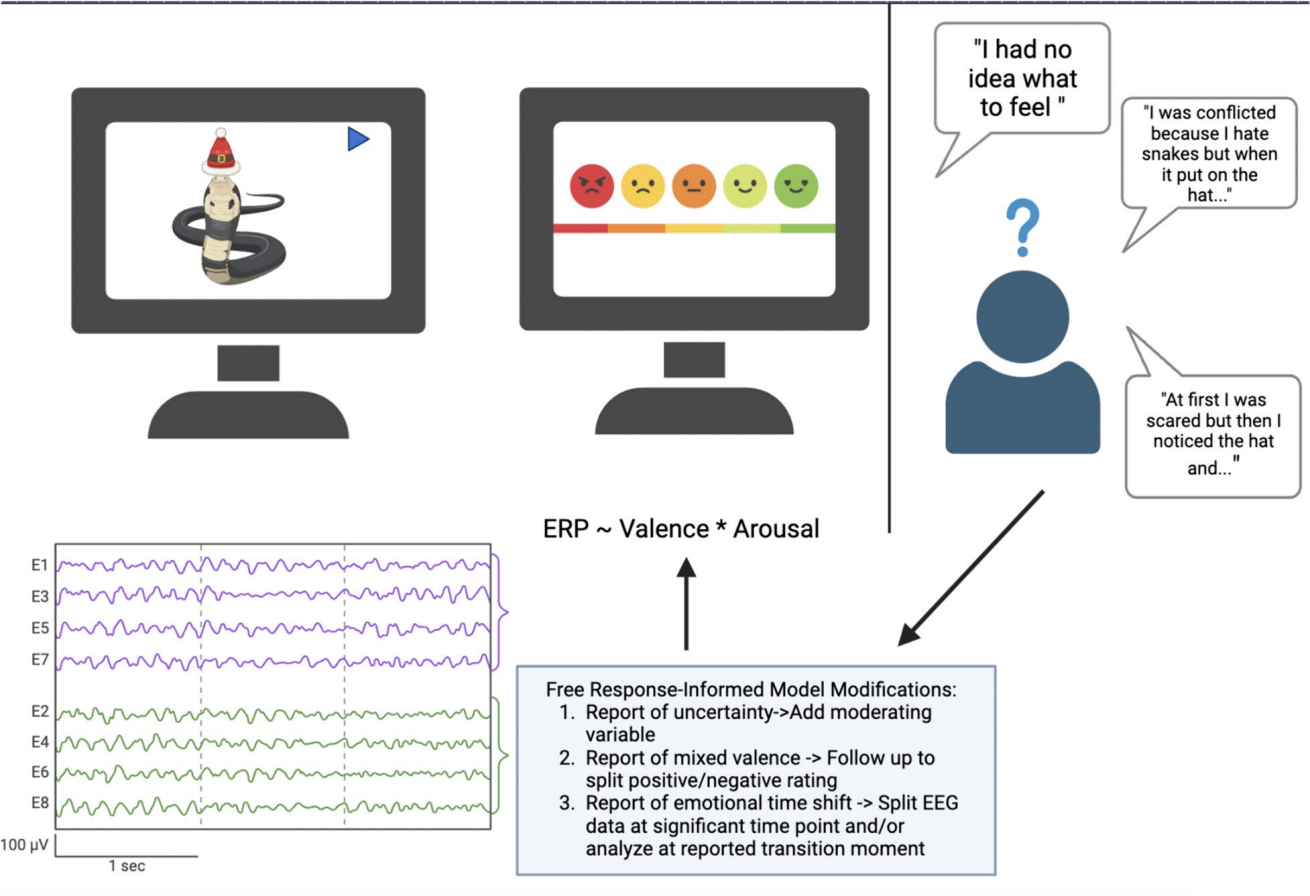
Using qualitative reports to fine-tune deep-imaging analyses. Emphasizing complex stimuli and intrasubject variability allows for a detailed exploration of affective responses, capturing blended, univalent, mixed, and ambiguous emotional states. Deep-imaging analyses using longer complex stimuli can investigate predictive features of affect in the same subject, taking full advantage of lab study control to collect detailed data on varying emotional states. Additionally, this set-up allows us to use qualitative responses to perform exploratory modifications to our original analyses, incorporating parts of the induced experience we did not anticipate. Figure created with BioRender.com

We can use stimuli to induce *strong and complex* feelings across the timecourse and, if sample size allows, across subjects. While this setup may lead to the fear that we “fail” to induce our intended state of study, or that we fail to anticipate some important aspect of what is induced, when formulating our hypotheses, we can take full advantage of the control lab studies give us in other ways. We can embrace lab experiments to collect as detailed data about the experience as possible based on what is observed. As an example with neuroimaging, we can perform intrasubject deep-imaging analyses using longer complex stimuli (see Fischer et al., 2024 for a detailed review). This approach allows us to investigate the predictive features of various aspects of affect (intensity, univalent vs mixed, ambiguity) due to the subject’s naturally varying emotional state, providing an expansive set of “trials” without concerns of averaging across subjects. Incorporating free-response from subjects, even after the study, can also allow for rich, qualitative data that can be used for exploratory follow-up analyses, potentially revealing insights we had not previously considered (see Fig. 2 for schematic).

Emphasizing complex stimuli and intrasubject variability allows for a detailed exploration of affective responses, capturing blended, univalent, mixed, and ambiguous emotional states. Deep-imaging analyses using longer complex stimuli can investigate predictive features of affect in the same subject, taking full advantage of lab study control to collect detailed data on varying emotional states. Additionally, this set-up allows us to use qualitative responses to perform exploratory modifications to our original analyses, incorporating parts of the induced experience we did not anticipate.

## Blends, Mixed Valence, and Uncertainty Force Affective Science to Evolve

At the opening talks of the Society for Affective Science’s 2024 meeting, Dr. Satpute emphasized how the data we collect, the experiments we perform, and our interpretations are strongly driven by our theoretical priors. This can lead us to a stalemate in adjudicating between different theories of affect and limit the growth and evolution of these theories. Our current theories of affect have almost exclusively been developed to explain univalent, non-blended, concrete feelings, and as such, the data we collect always fits. Studying and measuring messier aspects of affect forces these theories to evolve to explain what have traditionally been considered edge-cases, or even instances of measurement error (Russell & Carroll, 1999). As Wood & Coan (2023) point out, it is likely that seemingly competing emotion theories are at different levels of analysis, and not mutually exclusive. Understanding emotion blends, or what is occurring when subjects report uncertainty or confusion about their current emotional state, likely requires *both* levels of analysis (Ortony, 2022; Vaccaro et al., 2020). Having researchers with varying theoretical views study “messy” feelings will help us collaboratively make progress on various questions, such as the following:

- What aspects of affect are discrete vs dimensional? Can those discrete aspects blend?
- What univalent parts of affect (feelings, emotions, concepts) give rise to a reported mixed experience, and what temporal dynamics underlie that?
- What does it mean for a state to feel undifferentiated or to report uncertainty about your current state? How do interoceptive accuracy and concepts/labelling affect this?
- What aspects of affect are innate vs. culturally specific?

Paying attention to how complex emotional states are similar or different from less complex states in their behavioral consequences will also give us a better functional understanding of how affect can apply to real-world application. For example, accurate emotion recognition is vital for affective technologies, which aim to augment their support by adapting to the user’s emotional state (Seïler & Craig, 2016). Systems that cannot effectively measure the nuance in emotional scenarios, or can only classify incoming information as indicating a few select states, may respond ineffectively or harmfully (Braun et al., 2019; Chakriswaran et al., 2019; Christov-Moore et al., 2023). Insights gained from studying complex emotional states will be essential for creating systems that can effectively support users in various contexts, including education, mental health, and human–machine teaming (Wilson-Mendenhall & Holmes, 2023).

## The Future of Affective Science is Going to Get Messy—and That is Okay

My tentative proposals come from my specific background, as an affective neuroscientist who studies mixed emotions, and can be improved by those with other expertise. However, they offer a start towards richer data and expanded possibilities for collaboration. Importantly, while we will have to relinquish some level of experimental control, we can still maintain appropriate rigor. Feeling is interesting not because of its reducible simplicity but because of its chaotic and messy nature. If we want to advance the science of affect, we have to accept that our experiments, measures, and conceptualizations are going to get messier and that the mess is necessary.

## Additional Information

### Data Availability

No datasets were generated or analysed during the current study.
